# Endoscopic submucosal tunnel dissection using a novel bracing basket: An animal feasibility study

**DOI:** 10.1038/s41598-018-19203-6

**Published:** 2018-01-18

**Authors:** Min Min, Yan Liu, Xin Chen, Yiliang Bi, Wei Shen, Yang Xu

**Affiliations:** 1 Department of Gastroenterology and Hepatology, Affiliated Hospital of Academy of Military Medical Sciences, Beijing, 100071 China; 2 Department of Bioengineering and Therapeutic Sciences, University of California, San Francisco, CA 94143-0912 USA

## Abstract

The aim of this study was to evaluate the feasibility of a novel bracing basket for Endoscopic submucosal tunnel dissection (ESTD), which was developed for improved effectiveness and ease of use. This was a prospective randomized, comparative, experimental animal study carried out at a single center. The primary aim was to evaluate the efficacy of ESTD with a novel bracing basket, compared with conventional ESTD. The secondary aims were to assess the quality control of the procedures and adverse events. Twenty procedures (6 esophageal and 14 gastric) were performed in four pigs. All resections were completed as en bloc resections. The technical success rate was 100% for both techniques (bracing basket-assisted ESTD vs. conventional ESTD). The procedure times were similar, but the cutting speed was quicker with bracing basket-assisted ESTD in gastric (antrum:23.3 ± 2.2 mm^2^/min vs. 15.2 ± 3.2 mm^2^/min, body: 26.1 ± 1.3 mm^2^/min vs. 18.4 ± 2.0 mm^2^/min, p < 0.05). There was one bleeding in the bracing basket-assisted ESTD group and one perforation in the conventional ESTD group. Compared with conventional ESTD, the use of this basket has potential advantages. Comparison studies with larger gastric or colorectal lesions treated with conventional ESTD are needed.

## Introduction

Endoscopic submucosal tunnel dissection (ESTD) was first described in humans in 2010 by Inoue *et al*. for conducting peroral endoscopic myotomy^1^. Recently, it was proposed in esophageal and gastric for superficial cancer^2,3^. However, ESTD is a more challenging procedure than conventional ESD because of its technical difficulties, longer procedure time, and higher risk of adverse events such as perforation.

Despite improvements in equipment and ESTD techniques, depending on the location and shape of the tumor, each ESTD step may require different accessories, to achieve quality-controlled ESTD^4^. However, a major issue often encountered with large superficial tumors in the GI tract is maintaining an inadequate view during ESTD because the mucosa cannot be lifted, as in open surgery, which leads to difficulties in cutting the edges, especially the gravity side. Although mucosal flap formation improves visualization of the cutting area, it is still difficult to achieve, especially in colorectal ESD. To facilitate ESTD and make it more widely available, particularly for large superficial tumors in the GI tract, such as colonic laterally spreading tumors (LST), a simple and safe device is required. Inspired by the ERCP stone extraction basket, a new device makes for easily cutting the edge of the lesion to avoid complications, and it can easily determine the tunnel on both sides of the separation range; endoscopy can enter the tunnel for further separation.

We therefore designed a novel bracing basket for treating large superficial tumors in the GI tract. The aim of this study was to evaluate the feasibility of a novel bracing basket for Endoscopic submucosal tunnel dissection (ESTD), which was developed for improved effectiveness and ease of use.

## Materials and Methods

### Animals

**Figure 1:**
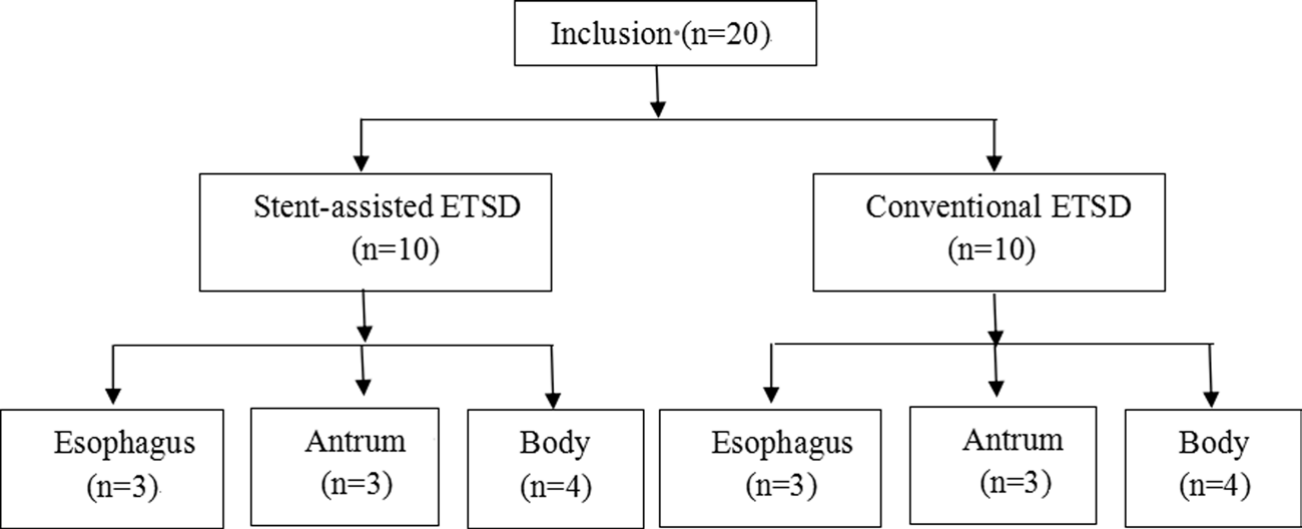
Flow diagram of the randomized procedures.

This is a comparative, experimental animal study carried out in the Affiliated Hospital of Academy of Military Medical Sciences, Beijing. The animals used for this study were healthy pigs weighing 20–25 kg. The animals received regular feeding until 48 hours before the procedure. Subsequently, they had a liquid diet until 12 hours before endoscopy. The procedures were conducted under general anesthesia. The order and site of the procedures were determined by randomization (sealing envelopes drawn at random). Each pig underwent basket-assisted ESTD and conventional in the gastric antrum, body and middle of the esophageal areas (Fig. 1). For examination of short term adverse events, the pigs were euthanized 24 hours after the completion of the procedures. All experimental procedures involving animals were performed in accordance with the guidelines for the National Care and Use of Animals approved by National Animal Research Authority of P. R. China. All of the experimental protocols were approved by the ethics committee of the Affiliated Hospital of Academy of Military Medical Sciences. All efforts were made to minimize animal suffering and to reduce the number of animals required.

### Newly Devices

**Figure 2:**
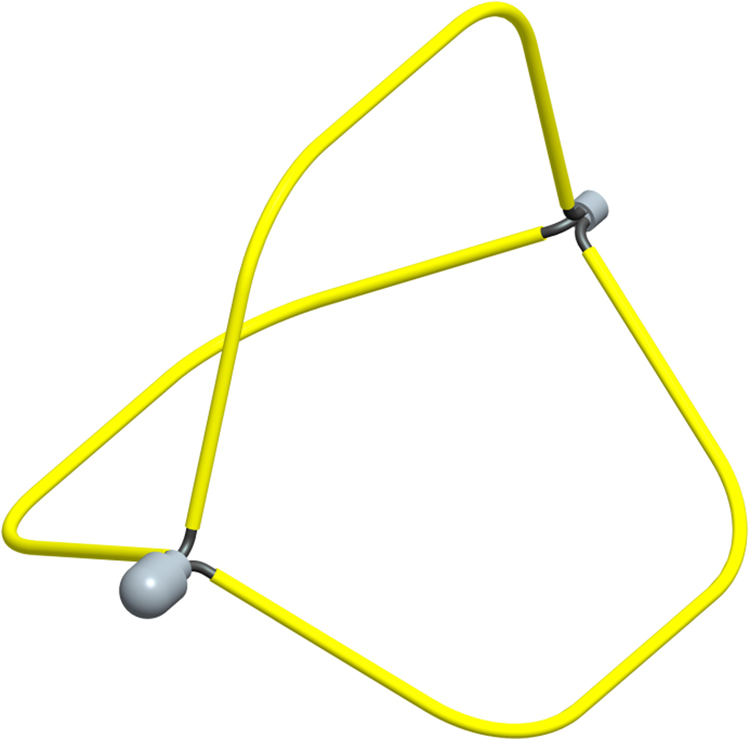
The novel bracing basket used in this study.

The new basket was designed by the authors and built by Leomed Company (Changzhou, China). A prototype of the bracing basket was developed (Fig. 2). The basket is made of nitinol and coated with an insulating material on the skeleton, to prevent accidental electric shock to human tissue during the ESTD procedure. It has external diameter of 25 mm and was eligible for a standard endoscope with a working channel width of 3.2 mm. When the basket is released, the top tube handle drive ram is moved forward, and the basket goes completely out of the sheath and returns to the original three-dimensional geometry of space. The basket is easily and quickly opened, to achieve the appropriate position in the submucosal tunnel. Depending on the lesion size and the endoscopic diameter, the basket was designed as 3 or 4 wires. When ESTD is completed, use grasping forceps, hooks or other auxiliary equipment and directly grasp the basket itself for withdrawal.

### Dissection technique

**Figure 3:**
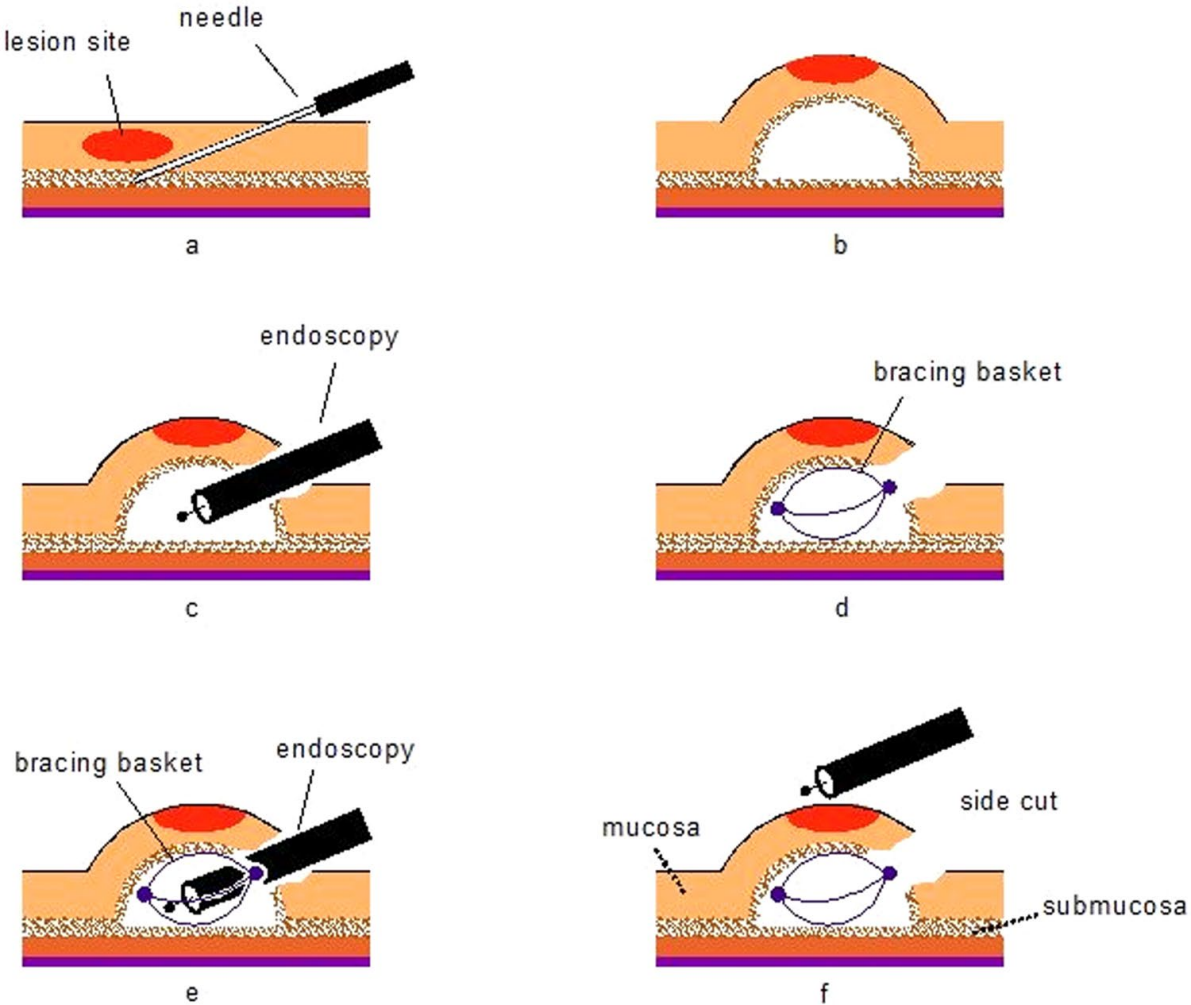
Summarizing the various steps of the procedure: (**a**) Injected mixed solution. (**b**) Submucosal lifting. (**c**) Create of the submucosal tunnel. (**d**) Push through the bracing basket into the tunnel. (**e**) Tunneling dissection and creation. (**f**) Cut the edge of the tunnel and final site of resection.

Three pigs underwent 20 removals of the stomach and esophageal mucosa; the lesions were virtual and target resected, with a specimen size of approximately 3 × 3 cm of mucosa, which were created by marking the border of the lesions electro-surgically using a DualKnife (Olympus Optical Co. Ltd, Japan). A single channel endoscope (EPX-4450; FUJINON Co., Tokyo, Japan; Olympus H180,Olympus Optical Co. Ltd, Japan) was used for all procedures. A disposable distal attachment cap (ND-201-11802, Olympus Optical Co. Ltd, Japan) was mounted onto the tip of the endoscope. An endoscopic injection needle (NM-4L-1, Olympus Optical Co. Ltd, Japan) was used to inject a mixed solution (a hypertonic saline solution mixed with diluted epinephrine (1:250,000), slightly stained with indigo carmine). All procedures were performed by a single endoscopist, who has sufficient experience in ESTDs, having performed more than 100 procedures with standard instruments. A submucosal injection was made. Once the mucosa was lifted, a horizontal mucosal incision was made (about one centimeter-sized, vertical), approximately 1 cm above the lesion and exactly in line with it to provide an opening into the submucosal space and thus create the tunnel’s entry point. The endoscope equipped with a transparent cap was inserted into the submucosa through the proximal opening to create the tunnel. Before circumferential cutting, the new device was inserted through the working channel of the endoscope and released into the tunnel. The traction was achieved by the basket, independent of the scope. Because the basket was positioned inside the tunnel, the corresponding edge of the tunnel could be tightly pulled during ESTD, independent of movement of the scope, enabling good visualization of the submucosal layer and cutting plane, especially the edges of the lesion (Fig. 3, video 1). The tip of the endoscope could be inserted in the basket, and enlarged the submucosal layer. The ICC 200 high-frequency generator (ERBE, Tubingen, Germany) was used for incision (endocut I, effect 3: duration 3, interval 3) and dissection (forced coagulation, effect 2, 50 W). Video 2 shows a representative case of the different devices (Dual knife, IT2 knife, SB knife) performed to cut the edge of the tunnel with using the bracing basket. Complete submucosal dissection was performed then the new basket was pulled out.

### Outcome evaluation

The primary outcome were defined the procedure time and speed, the second outcome were completement of dissection, procedural adverse events (bleeding and perforation).

### Procedural measures

The completion time of each resection was documented, and the dissected specimens were retrieved and evaluated for completeness (en bloc resection). The time for complete dissection including marking of lesions, initial injection for mucosal incision, and removal of resected specimen. Cutting speed was measured as the desection area divided by the entire cutting time (duration from start of the mucosal incision to completion of resection). Completeness of dissection was assessed by endoscopic and macroscopic inspections of the resection site for any remaining coagulation markings. Technical success was defined by a resection performed entirely by the dissection technique selected, that either basket-assisted or conventional, as described below. Adverse event was including perforation and bleeding. The perforation was diagnosed during endoscopic and histopathological examinations. Significant bleeding was defined as any use of hemostatic clip.

### Statistical analysis

A Mann-Whitney U-test was performed for the analysis of the primary objective criteria, such as procedure time and cutting speed, and Fisher’s exact test was used for comparison of adverse events between the two modalities. *P* < 0.05 was considered significant. Statistical analysis was performed with SPSS version 19.0.0.

## Results

Two pigs were used, as part of this study, giving a total of twenty ESTDs. The sites of resection were gastric antrum (Greater curvature), body (Posterior wall of lower body) and middle of esophagus in each pig. By using the approach as stated, all experiments were successfully performed, with areas totally resected. The en bloc resection rate and the technical success rate were 100%, respectively, in both groups. The basket-assisted ESTD group used only one basket per lesion. The characteristics of the various procedures are summarized in Table 1. The median procedure times were 25.4 ± 3.5 min vs. 26.9 ± 2.2 min in the esophagus, 30.2 ± 4.5 min vs. 32.4 ± 2.2 min in the gastric antrum, and 28.0 ± 3.2 min vs. 31.3 ± 2.6 min in the gastric body (bracing basket-assisted ESTD vs. conventional ESTD, respectively).

**Table 1:** Statistical Analysis of Procedural Data.

Variables	basket-assisted ESTD (n = 10)			Conventional ESTD (n = 10)		
	Esophagus	Antrum	Body	Esophagus	Antrum	Body
Procedure time,min	25.4 ± 3.5	30.2 ± 4.5	28.0 ± 3.2	26.9 ± 2.2	32.4 ± 2.2	31.3 ± 2.6
building tunnel time, min 5.1 ± 1.1	9.2 ± 2.0	7.2 ± 1.2		5.5 ± 1.0	8.4 ± 3.2	10.7 ± 4.6
Cutting speed, mm^2^/min	6.2 ± 1.1	23.3 ± 2.2*	26.1 ± 1.3*	8.3 ± 1.1	15.2 ± 3.2	18.4 ± 2.0
Completeness rate, %	100	100	100	100	100	100
Bleeding frequency	0	1	0	0	0	0
Perforation	0	0	0	1	0	0

Data are presented as mean ± SD.

*p < 0.05 compared with the Conventional ESTD.

The median cutting speed was significantly shorter for the bracing basket-assisted ESTD than the conventional ESTD in gastric (antrum: 23.3 ± 2.2 mm^2^/min vs. 15.2 ± 3.2 mm^2^/min, body: 26.1 ± 1.3 mm^2^/min vs. 18.4 ± 2.0 mm^2^/min, p < 0.05). No significant differences were observed in the occurrence of adverse events between the two groups. However, one microperforation occurred in one esophageal case (conventional ESTD group), during the submucosal space was being dissected, the perforation were no bigger than a few millimeters, and could be treated conservatively, There were no gastric perforations. During the ESTD, several bleeding episodes occurred, most of them was successfully treated with Dual knife in soft coagulation mode. One severe arterial bleeding incident occurred during the basket-assisted ESTD procedure and could be controlled by use of hemostatic clip. All the pigs were euthanized 48 h after the completion of the procedures and necropsy showed the microperforation was covering the longitudinal fibers of the muscularis in the findings.

## Discussion

Here, we reported the results of an animal feasibility study for ESTD using a novel self-expanding basket, which was inspired by the ERCP stone extraction basket. Although the procedure time of the bracing basket-assisted ESTD was similar to conventional ESTD, the cutting speed was quicker, and all lesions were dissected en bloc.

In ESTD, it is important to maintain a good operative view during treatment. Achieving a good operative view is not easy because only an electrosurgical knife can be passed through the single working channel. Visualization of the submucosal layer is a promising technique to reduce the duration of the procedure and the development of associated complications^5,6^. Adequate tissue tension and good visibility of the tissue to be dissected are very important for safe and effective dissections and can be achieved by traction of the tissue to be dissected. Traction by adjunctive devices requires additional materials and time; specialized, expensive, or even very large devices may be required^7–9^.

In our study, we conclude some experiences to using the basket. In the esophagus ESTDs, because the space of the esophagus is limited and lacks a serosal layer, and there is only a thin membrane outside the MP layer, using the basket was technically challenging and has potential perforation risk. Although the attachment of the mucosal and muscular layer is loose, a submucosal tunnel can easily be created and a basket can also easily provide the traction in the tunnel. To avoiding perforations, based on our experience, when after completion of the tunnel, the basket could inserted into the tunnel and aim at shorten the time of cutting edge of the tunnel. In the gastric and esophagus ESTDs, the basket was inserted into the tip of the working channel, and the biopsy forcep was pushing it down through the working channel and released it into the tunnel. The new workflow of the procedure may be prolong compared with the conventional ESTD. Furthermore, in some occasions, the basket need adjust its position and we used the biopsy forcep to grasp and move it. Although this is conventient, it is still time-costing. In our study, owing to the target specimen size of approximately 3 × 3 cm of mucosa, we chose the diameter of 25 mm basket with 3 wires. Clinically, if the lesion is larger, we may chose the 4 or 5 wires basket to keep the traction, this could be a potential to impede endoscopic therapy owing to the many wires in the tunnel. In the future, we will design the basket which has more tension but less wires to solve this problem.

In our study, this novel basket had a number of advantages. First, traction with the new basket required less consideration of gravity than traditional methods and patients needed only to be placed in positions that allowed for the accumulation of air and easy operation of the endoscope. It can not only for the section of the lateral edges of the tunnel but also to help in creating the tunnel. Second, during ESTD, the accessories may need to be frequently changed, due to the use of various accessories, which is time-consumingand expensive^10–12^. Use of this new basket made ESTD easier and may have reduced the procedure time for submucosal dissection, although this difference was not significant. However, it should be noted that we released the basket and adjusted its position, which led to longer procedure times. This difference in procedure time may also be because the endoscopist already possessed experience with ESTD but not with the new basket. Despite this issue, cutting speeds were quicker with the basket. Third, the basket can provide better submucosal exposure. The precise dissection of the submucosal layer is the most important step for preventing perforation, and the electrosurgical knife should always aim for the submucosal layer during dissection. Moreover, various methods using clips and a thread have been attempted in esophageal or gastric ESD^13–15^; however, the majority of these methods has not been tested in colorectal ESD. Because the colorectal is much thinner than the gastric wall, the risk of perforation is considerably greater in colorectal ESD than in gastric ESD. Various other adjunctive methods have been developed to control and strengthen the traction force in ESD for colorectal cancer lesions, but most are complicated and have some limitations when performed in the deep colon^13,16^. Unlike the use of the clip-band technique or the clip-flap method, with this new basket, it is possible to adjust the direction and intensity of traction during dissection. Further prospective studies are warranted evaluate the efficacy and safety of the basket in colorectal ESTD.

There are some limitations that need to be considered for this study. First, improvements to the basket design are required before it can be advocated for routine use in ESTD. The choice of an appropriate basket size is very important before the procedure. Second, significant fibrosis can sometimes occur in the submucosal layer and cause difficulties in insertion of the new basket. Third, although in this study we were not confronted with massive bleeding in the tunnel; its management could be more challenging because the basket may reduce more working space. Fourth, use of the new basket also has not been evaluated in some locations (e.g. angulus, fundic stomach) because creating a tunnel would probably be more difficult in these locations.

In conclusion, the novel bracing basket-assisted ESTD facilitated manipulation of the endoscope and treatment device, compared with conventional ESTD, the use of this basket has potential advantages. As the present results were based on the experience of a single endoscopist, who performed a limited number of animal studies, in the next step toward clinical application, the utility of this new device will need to be verified in humans. Comparison studies with larger gastric or colorectal lesions treated with conventional ESTD are needed.

## Electronic supplementary material

video 1

video 2
